# Bridging Gaps in Peripheral Nerves: From Current Strategies to Future Perspectives in Conduit Design

**DOI:** 10.3390/ijms24119170

**Published:** 2023-05-24

**Authors:** Elena Stocco, Silvia Barbon, Aron Emmi, Cesare Tiengo, Veronica Macchi, Raffaele De Caro, Andrea Porzionato

**Affiliations:** 1 Section of Human Anatomy, Department of Neuroscience, University of Padova, 35121 Padova, Italy; elena.stocco@unipd.it (E.S.); silvia.barbon@unipd.it (S.B.); aron.emmi@unipd.it (A.E.); veronica.macchi@unipd.it (V.M.); andrea.porzionato@unipd.it (A.P.); 2 Department of Cardiac, Thoracic, Vascular Sciences and Public Health, Padova University Hospital, Via Giustiniani, 2, 35128 Padova, Italy; 3 L.i.f.e.L.a.b. Program, Consorzio per la Ricerca Sanitaria (CORIS), Veneto Region, 35128 Padova, Italy; 4 Foundation for Biology and Regenerative Medicine, Tissue Engineering and Signaling-TES, Onlus, 35030 Padova, Italy; 5 Plastic Surgery Unit, Department of Neuroscience, University of Padova, 35121 Padova, Italy; cesare.tiengo@unipd.it

**Keywords:** peripheral nerve injury, autograft, allograft, hollow nerve conduits, wall functionalization, luminal fillers

## Abstract

In peripheral nerve injuries (PNI) with substance loss, where tensionless end-to-end suture is not achievable, the positioning of a graft is required. Available options include autografts (e.g., sural nerve, medial and lateral antebrachial cutaneous nerves, superficial branch of the radial nerve), allografts (Avance^®^; human origin), and hollow nerve conduits. There are eleven commercial hollow conduits approved for clinical, and they consist of devices made of a non-biodegradable synthetic polymer (polyvinyl alcohol), biodegradable synthetic polymers (poly(DL-lactide-ε-caprolactone); polyglycolic acid), and biodegradable natural polymers (collagen type I with/without glycosaminoglycan; chitosan; porcine small intestinal submucosa); different resorption times are available for resorbable guides, ranging from three months to four years. Unfortunately, anatomical/functional nerve regeneration requirements are not satisfied by any of the possible alternatives; to date, focusing on wall and/or inner lumen organization/functionalization seems to be the most promising strategy for next-generation device fabrication. Porous or grooved walls as well as multichannel lumens and luminal fillers are the most intriguing options, eventually also including the addition of cells (Schwann cells, bone marrow-derived, and adipose tissue derived stem cells) to support nerve regeneration. This review aims to describe common alternatives for severe PNI recovery with a highlight of future conduits.

## 1. Introduction

Successful management of peripheral nerve injuries (PNI) is an intriguing challenge in both surgery and research science, and improvements are imperative; over 5 million new cases of PNI are expected worldwide every year [1]. These kinds of lesions, much more frequent and undervalued than spinal cord injuries [2], occur because of several events (rushing, stretching, compression, avulsion, and total or partial nerve transection) and, despite the increase in knowledge of the mechanisms of injury and regeneration, a full functional recovery is still unsatisfying in most patients undergoing surgical reconstruction [3,4]. The loss of the nervous supply directly affects muscular function due to the interruption of neuromuscular communication. In turn, this event causes a decrease in muscle activity, which triggers an adaptive response to tissue remodeling marked by the degradation of muscle contractile proteins. Consequently, a decrease in mass, fiber diameter, and production of force, which together characterize muscle atrophy, can be observed [5].

Sensory and motor function impairments are responsible for lifelong disability with a permanent impact on the person’s ability to perform the activities of daily living, including the return to work, and a remarkable decrease in patient’s quality of life [4]. It follows that treatment is essential. In clinical practice, the approach to PNI strictly depends on injury severity [6] in turn influencing the degree of recovery. While neuropraxia (compression/mild crush injury with Schwann cell sheath involvement, maintaining the integrity and continuity of the axons and connective tissue) and axonotmesis (axon and myelin sheath integrity loss, with maintenance of the outer layers of connective tissue and nerve anatomical shape) are more often associated with promising outcomes after treatment, neurotmesis corresponds to total disruption of the axons and its surrounding layers with lower recovery outcomes depending on both the gap width and the intervention time [2].

In case of sharp lesions without substance loss or gaps shorter than 3 mm, direct repair (i.e., neurorrhaphy) is commonly performed, also supported by supplementary procedures including protection of the nerve with natural/synthetic wraps to reduce inflammatory/immunologic reaction at the suture site [7,8]. Differently, in damages with severe interruption of nerve continuity when tensionless coaptation is not possible, bridging the proximal and the distal stumps is required. To this purpose, several grafting methods are available in clinical practice, including autografts, allografts, and hollow nerve conduit positioning. After considering the physiological mechanism associated with lesioned nerve regeneration, the aim of this review is to provide an overview of current strategies for treatment of such conditions, also shading a light over future perspectives in vanguard device development.

## 2. Physiology of Lesioned Nerve Regeneration: Wallerian Degeneration and Bands of Büngner

Following nerve injury, physiological and cellular events occur at both the proximal and the distal stumps. While at the proximal end axon degeneration takes place up to the first Ranvier’s node, at the distal stump non neuronal cells trigger an inflammatory response which in turn initiates a process called Wallerian degeneration, consisting of axonal disintegration. Briefly, after nerve transection, the cytoskeleton and the axon cell membranes are rapidly (within 1 h from injury) fragmented into small debris and the Schwann cells, associated with the axons, change their phenotype, expelling their myelin sheaths and becoming phagocytotic [4]. Contextually, the Schwann cells are also responsible for other different mechanisms, including induction of regeneration associated genes and secretion of extracellular matrix (ECM) molecules (supportive function), trophic factors (axons growth promotion), and cytokines/chemokines (immune cells recruitment). Immune cells play a fundamental role during PNI repair/regeneration. Neutrophils and macrophages (endoneurial and circulating) are involved in clearing debris from the injury site by phagocytosis; moreover, they also induce activation of the monocytes, thus differentiating into new macrophages that in turn concentrate at the lesion site, furtherly supporting phagocytosis. Following the inflammation phase, the activated Schwann cells undergo proliferation, forming new myelin sheaths and reinnervating the target tissue [9,10]. Specifically, Schwann cells distal to the injury site play a key role in driving axon regrowth; they dedifferentiate and form longitudinal cell strands appearing as aligned tubular guidance structures called bands of Büngner. These structures, appearing as hundreds of pro-regenerative microchannels, guide axonal regeneration in a pathway-like manner. Thus, they can be compared to a natural scaffold that promotes a targeted reinnervation [11]. Whether regenerating axons accidentally leave the bands of Büngner, elongation stops with consequent formation of a painful neuroma. Even though the mechanism under the bands’ formation remains largely unknown, its fundamental role in axonal regeneration is indisputable [12].

## 3. Current Bridging Strategies

### 3.1. Autografts

**Figure 1:**
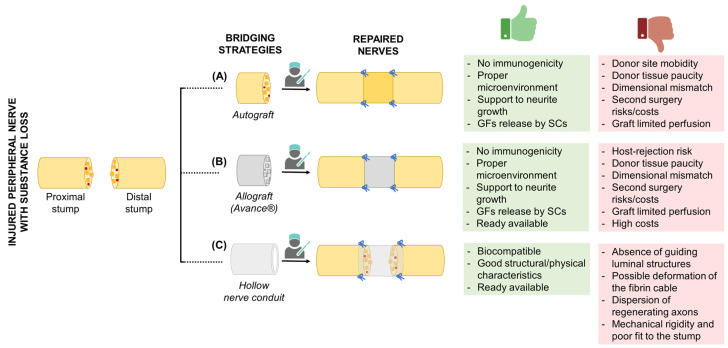
Bridging strategies for the repair of injured peripheral nerves with substance loss. For each strategy, including (**A**) autograft, (**B**) allograft (Avance^®^), and (**C**) hollow conduit positioning, the figure reports the specific advantages (green box) and limitations (red box). GF, growth factors; SCs, Schwann cells. The Figure was partly created with BioRender.com (accessed on 17 May 2023).

Autograft implantation consists of replacing an injured peripheral nerve with a nerve autograft; sural nerves, medial and lateral antebrachial cutaneous nerves, superficial branches of the radial nerve, dorsal cutaneous branches of the ulnar nerve, superficial and deep peroneal nerves, intercostal nerves, and the posterior and lateral femoral cutaneous nerves are mainly used for this purpose [13] and, to date, this approach is considered the “gold standard” in clinical practice because it minimizes immunological reactions while providing a proper microenvironment for nerve regeneration. Specifically, the native nerve physical structure and the Schwann cells’ basal lamina within the graft play a fundamental role in supporting neurite growth [14], behaving as a sort of template for regeneration. Moreover, the graft-resident Schwann cells also release neurotrophic factors that support repair [15]. Unfortunately, donor site morbidity (including donor site neuroma and associated pain), paucity of donor tissue, eventual mismatch between the donor/recipient nerve (both in terms of size and alignment), and the risks/costs of the second surgery are non-negligible drawbacks associated with autografts [13,16,17,18]. Additionally, Schwann cells within the graft may undergo necrosis once transplanted as a consequence of a possible limited perfusion from the surrounding vasculature (the graft is too thick, and revascularization cannot reach the center of the graft; the graft is too large and surrounding vasculature cannot provide sufficient nutrients and oxygen to the entire graft), resulting in poor functional repair [19] (Figure 1A). All these critical issues have prompted movement towards the identification of possible therapeutic alternatives to improve the prognosis for patients.

### 3.2. Allografts

An alternative to autografts is represented by allografts consisting of nerve grafts from cadaver donors that, once adequately processed, can be stored in tissue banks until use. References to their use in clinical practice have been reported since the 1960s, when donated nerves were preliminarily treated with freezing and irradiation before use. However, despite avoiding the autograft-related issues, the allografts prepared in this way face the risk of host rejection, mainly directed against the Schwann cells and myelin sheaths of the graft, in turn precluding axonal regeneration [20,21]. Several attempts were made to improve allografts’ performances in vivo, focusing on immunogenic response reduction strategies (major histocompatibility complex (MHC) matching [22] and blood-type (AB0) matching) [23]; however, immunosuppression was revealed to be required up to 18 months post-implantation, in turn increasing patients’ susceptibility towards opportunistic infections that, in the poorer prognosis, may also lead to tumor formation [24,25,26]. Cryopreservation, lyophilization, freezing/thawing, and chemical treatments were included among the several strategies performed to achieve a “safe” allograft [27], but only decellularization led to interesting results with the development of Avance^®^ (AxoGen Inc., Alachua, FL, USA; human origin), first commercialized in 2007 after a journey from the bench to the bedside of about 20 years [4]. Avance^®^ results from the combination of two protocols developed by Hudson et al. [28] and Krekoski et al. [29], consisting of nerve treatment with mild detergents (Sulfobetaine-16, Triton X-200, and Sulfobetaine-1) and chondroitinase, respectively. Interestingly, preoperative chondroitinase treatment proved to boost axonal penetration into the acellular grafts [29].

Despite appealing outcomes, limitations are also associated with allografts; caution must be applied to the use of long grafts with larger diameters [30,31]. Moreover, allografts require cadaveric donors to harvest tissues and isolation/graft preparation is highly costly [27] (Figure 1B). The availability of an off-the-shelf, fully synthetic nerve conduit would represent an intriguing alternative to the field, meeting the requirements of both the patient and the surgeon.

### 3.3. Hollow Nerve Conduits

To overcome issues and challenges related to autografts and allografts, different nerve conduits have been developed aiming to achieve safe, on-the-bench, alternatives. These devices, once positioned through epineural suture between the proximal and the distal stump, restore nerve continuity while guiding nerve regeneration. To date, the US Food and Drug Administration (FDA) has approved different nerve conduits for clinical use, fabricated through different techniques: rolling of a mesh, precipitation on a rotating mandrel, or dip-coating of a rotating mandrel [32]. Regarding the biomaterials they are made of, both natural polymers (collagen type I, extracellular matrix from porcine small intestine submucosa, chitosan) and synthetic polymers (polyvinyl alcohol (PVA), polyglycolic acid (PGA), poly(D,L-lactide-co-ε-caprolactone) (PLCL)) are included. Except for the PVA-based conduit (SaluTunnel, non-biodegradable), the devices’ degradation profiles range from 3 months (NeuroTube, PGA) to 48 months (NeuraWrap, Neuragen, collagen type I) [7,33,34,35,36,37,38,39,40].

Even though these products are approved for clinical use, as they do not cause cytotoxic reactions, irritation, sensitization, acute/chronic toxicities, genotoxicity, or hemolysis, and show good structural/physical characteristics (suture retention, no kinking) as well as adequate porosity and permeability, they still fail to promote full functional recovery in many patients, especially in the case of long gap injuries (>3 cm) [41]. Evidence demonstrates that they may be unable to regenerate and recover complete nerve function, and also perform inferiorly compared to autografts. The absence of luminal structures (biological/biochemical/biophysical cues) to guide ordered axon and Schwann cell regeneration was highlighted as a possible contributing cause for their failure in long gap PNI treatment. The longitudinal fibrin cables (“blood clot”) forming from the fluid secreted by the damaged nerve ends are often inadequate or disrupted, thus not providing for that fundamental microarchitecture guiding Schwann cells within the injury site. Mechanical forces during regeneration may be responsible for a deformation of the fibrin cable, taking on the shape of an “hourglass”, with consequent limitation of the regeneration area [4,42]. In turn, dispersion of regenerating axons as well as inappropriately targeted re-innervation likely occurs [43,44,45]. Moreover, mechanical rigidity, a poor fit to the end-stump, and a lack of customization are among the weaknesses of the current devices (Figure 1C).

None of the commercially available guiding conduits (Table 1) [7,33,34,35,36,37,38,39,40,46] guarantee fully satisfactory results in neuronal and muscular regeneration following neurotmesis. All these issues are boosting research into material sciences, biotechnologies, and tissue engineering towards the identification of effective alternatives overcoming current devices limitations.

## 4. Future Directions in Nerve Conduits

### 4.1. Promising Conduits Design

From biopolymers and synthetic polymers to blends, there are numerous material options for nerve conduit fabrication due to consciousness that the intrinsic characteristics of materials can affect the regeneration outcome. However, topography can also significantly modulate the ability of the nerve to regenerate across the gap [47], with an effect over the attachment, spreading, growth, differentiation, and transcription profile of the cells [48].Thus, apart from the already-discussed traditional conduits (hollow/non-porous), four different nerve guide designs could be recognized as promising: (i) porous, (ii) grooved, (iii) multi-channel, and (iv) nerve conduits with fillers (fibers or hydrogels) [49].

**Figure 2:**
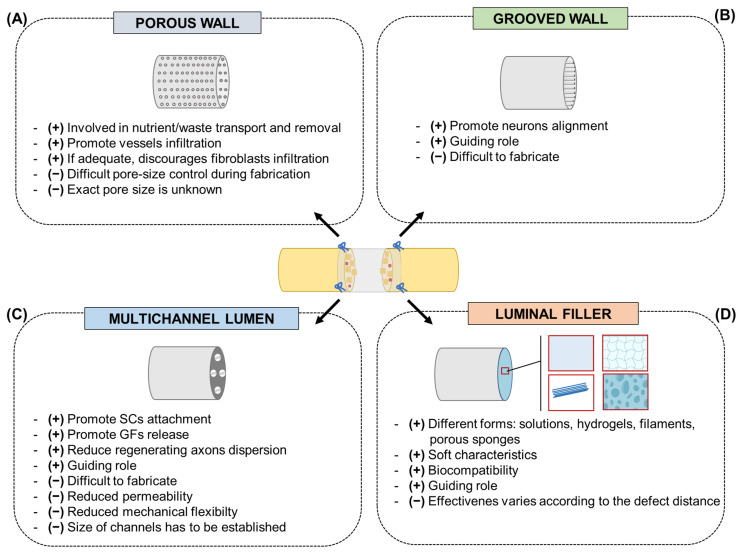
Future conduit designs with pros (+) and cons (−). (**A**) Porous wall; (**B**) grooved wall; (**C**) multichannel lumen; (**D**) luminal filler. GF, growth factors; SCs, Schwann cells. The Figure was partly created with BioRender.com (accessed on 17 May 2023).

Porous wall. The porosity of a scaffold could have effects on cell viability and proliferation, as involved in dynamic events such as nutrient transport, removal of waste products, and blood vessel infiltration. Contextually, adequate porosity also discourages cell infiltration (fibroblasts) that may obstruct axon extension. Porosity and pore size are often dependent on the method of scaffold fabrication [50]; despite the optimal range, recognized to be in the range of 5–30 μm [51], information on the exact pore sizes of the scaffolds is lacking, thus limiting their translation to future nerve guide designs. Pore size control during fabrication is also difficult (Figure 2A) [52].

Grooved wall. Cells were shown to migrate or grow in a certain direction dictated by the physical morphology of the seeding surface. Linear microgrooves, obtained by microfabrication techniques (lithography), are the most effective to this purpose, but particular attention is required for the dimensions of the aligned pattern [53]. Grooves with widths and spacings of 10–20 µm are recognized to be preferred by the Schwann cells even though, in general, it has been reported that topographic features comparable with cell ranges (10–50 µm) are promising [54]. Groove depth can also affect surface/cells interaction. To support neurite alignment, depths ranging from 1 to 4 µm are adequate; conversely, sizes inferior to 300 nm were revealed as less favorable [55,56,57]. Difficulties in fabrication are the main limit in the development of grooved conduits, and it is undeniable that this also depends on the material used (Figure 2B).

Multichannels and fillers. Together with wall design, luminal modifications are likewise gaining importance as an intriguing strategy for nerve conduit functionalization. Typically, providing for a direction-instructive stimulus, they serve as secondary scaffolds within the nerve conduits, hypothetically allowing for increased neurite and Schwann cell outgrowth/proliferation. Moreover, the eventual presence of biochemical stimuli (neurotrophic peptides and/or growth factors) within may furtherly boost physiological mechanisms associated with injury recovery [58]. Potentially, axonal regeneration can be enhanced if the bridging structure is designed to direct and organize the longitudinal formation of the nerve cable [59]. Architectural modifications of the lumen can be made at macroscale (multi lumen, rolled, and hydrogel), microscale (microgrooves, microfilaments, or microfibers and microporous), and nanoscale (nanoimprints) levels [19,53]; the scale of the lumen architecture strictly depends on the technology used to fabricate the tubular constructs.

Multiple channels promote the attachment of Schwann cells and the release of growth factors while reducing the dispersion of regenerating axons. A multichannel conduit dividing the large hollow tube into several small channels that mimic the fascicular structure of native nerves is recognized as adequate to ensure the guiding role of the conduit [60,61]. In terms of fabrication methods, various technologies have been adopted, including solvent or thermally induced phase separation, injection molding, electrospinning, and 3D printing [62]. However, multichannel conduits seem to reduce permeability and mechanical flexibility, thus not providing more significant advantages than hollow conduits [63]. Attention must also be paid to multichannel size as macro-sized lumens may fail in providing adequate physical cues to direct axon outgrowth [64] (Figure 2C).

Regarding luminal fillers, different natural polymers, including fibrin, collagen, laminin, and agarose are often used in the form of solutions, hydrogels, filaments, and porous sponges; by virtue of their soft characteristics and biocompatibility, they can adequately behave as a regenerative guidance for PNI repair, supporting nerve conduit function [42]. In parallel, synthetic polymers can also be introduced into nerve conduits as aligned fibers/filaments; among materials, polyamide, polyacrylonitrile-co-methylacrylate, polyglycolic acid, poly-L-lactic acid, and poly(lactic-co-glycolic acid) are included [65]. Though many luminal fillers have been reported, their effectiveness can vary according to the specific distance of the nerve defect. In the perspective of clinical translation, the fundamental requirement for them is to be readily manufactured and easily introduced within the conduit [42] (Figure 2D).

Many of the materials used as fillers aim to reproduce an ECM-like environment in terms of biologic stimuli and/or ultrastructure. Interestingly, a new class of compounds that can spontaneously give rise to stable α-helix, β-sheet, or random coil, in turn organizing into 3D aggregation states resembling ECM ultrastructure, is represented by Self-Assembling-Peptides (SAPs). Due to their specific behavior, their reveal was important to the field of nanotechnology and biotechnology for possible application in tissue engineering [43,66]. Additionally, the SAPs can also be modified with functional specific motifs holding great clinical promise for PNI repair (e.g., IKVAV, sequence: isoleucine–lysine–valine–alanine–valine; RGD, sequence: arginine–glycine–aspartic acid; YIGSR, sequence: tyrosine–isoleucine–glycine–serine–arginine) [67,68,69,70]. Considering the potential of SAPs and SAPs-conjugates, they may enhance the neuro-conductive microenvironment of conduits.

### 4.2. Electroconductive Conduits

**Figure 3:**
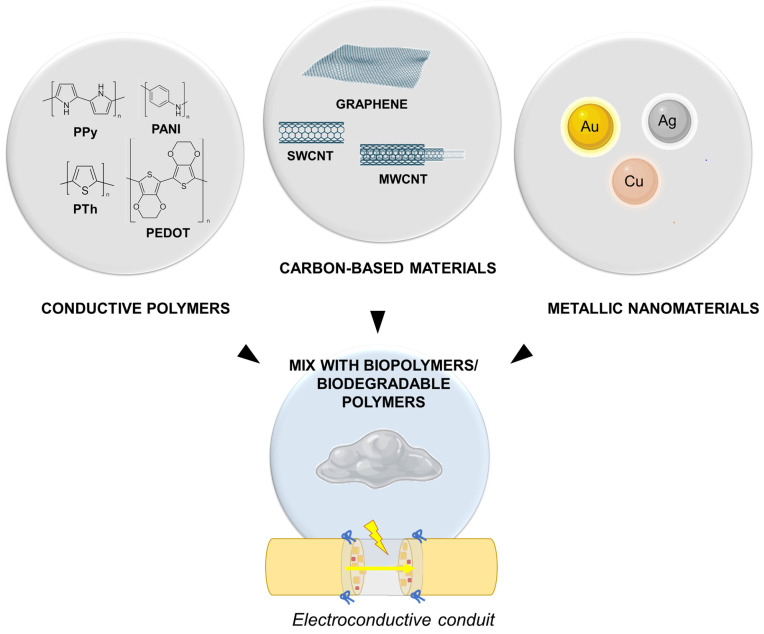
Electroconductive conduit development. Conductive polymers, carbon-based materials, and metallic nanomaterials can be mixed with different biopolymers or biodegradable polymers to obtain effective electroconductive conduits. PPy, polypyrrole; PANI, polyaniline; PTh, polythiophene; PEDOT, poly(3,4-ethylenedioxythiophene); SWCNT, single wall carbon nanotube; MWCNT, multi wall carbon nanotube; Au, gold; Ag, silver; Cu, copper. The Figure was partly created with BioRender.com (accessed on 17 May 2023).

Both in laboratory and clinical settings, electrical stimulation emerged as an interesting strategy to support the repair and regeneration of different tissues (muscle, tendon, nerve, and articular tissue) through the regulation of cellular activities (cell adhesion, proliferation, cell migration, protein production) [71]. Specifically, referring to peripheral nerves, electrical stimulation employing electrically conductive substrates was shown to effectively promote neurite and axon growth [72,73], and “electroceutical” therapies, referring to those approaches based on electrical impulse delivery for neurologic disease treatment, arose as a hot topic of research. In the case of sharp nerve transections, it was shown that the application of low-frequency (20 Hz or less) electrical stimulation just after primary surgical repair effectively enhanced functional recovery after PNI, in both animal models of disease and in patients [74]. Thus, this evidence triggered further research into electroconductive nerve conduits to ameliorate outcomes also in treatment of severe PNI. As a major part of conductive conduits lacks in flexibility and/or permeability, conductive hydrogel development based on the incorporation of conductive materials (conductive polymers, carbon-based materials, metallic nanomaterials) are an interesting option to resort to, as the show porosity and softness while exhibiting conductance [75] (Figure 3).

Polypyrrole (PPy), polyaniline (PANI), and polythiophene (PTh) and its derivatives, such as poly(3,4-ethylenedioxythiophene) (PEDOT), are among the most studied conductive polymers in the biomedical field [76]. The electroconductivity of conduits based on them is intrinsic and attributable to the structure and electrical properties of the polymers they are made of, showing both the chemical properties of organic polymers and the electrical properties of metals, in turn supporting electrical signal propagation [77]. Briefly, PPy has excellent electrical behavior, also displaying cell and tissue compatibility; it is easy to synthesize (with an easily modifiable surface) and it is inexpensive, thus standing out as appealing for tissue engineering purposes. Its drawbacks include poor solubility and degradation profile [78]; thus, pure PPy is not adequate for conduit fabrication. Several biodegradable polymers (polylactic acid (PLA), poly(lactide-co-epsilon-caprolactone (PLCL), and polycaprolactone (PCL)), have been used for hybrid conduit development with PPy [79]. Similar to PPy, PANI has an extraordinary ability to conduct electricity; it is biocompatible and also has low toxicity; moreover, it is easy to prepare and environmentally stable. However, even in this case, low solubility and processability limited its use, leading to development of PANI-based composites and blends [80]. PTh shows PPy-like characteristics; the most attractive derivative is PEDOT, which is distinguished by its electrochemical and environmental stability and stronger electrical conductivity than PTh. The introduction of electric polymers into biopolymers (cellulose, chitosan, hyaluronic acid, gelatin, etc.) has already been proved an efficient strategy for supporting nerve regeneration [81].

Together with conductive polymers, carbon-based materials including inorganic graphene and carbon nanotubes (CNTs) display a high range of applications in nerve conduit set-up. Graphene has a honeycomb planar structure made of one or more layers of carbon atoms. Together with its derivatives, it has great potential in PNI recovery due to high safety, chemical stability, high electrical conductivity, as well as a high surface-to-volume ratio. Compared to conductive polymers, graphene-based materials are distinguished by flexibility and mechanical strength, allowing them to excel at neural tissue engineering [82]. Among graphene derivatives, CNTs are included. These hollow cylindrical nanostructures, made of rolled graphene sheets in a single wall layer (SWCNTs) or in multiwalled layers (MWCNTs), elicited significant interest in the biomedical field due to peculiar physicochemical properties that, together with electroconductivity and high mechanical strength, include nanoscale morphology and tunable surface chemistry able to support cell adhesion, growth, and the differentiation of neurons [83,84]. Due to scant dispersion in aqueous medium, tailored chemical modification of CNTs represents a fundamental strategy to guarantee their efficient dispersion within a polymer phase. This strategy also allows for aggregation preventing mitigating possible toxicity issues [85].

Metallic nanomaterials are recognized to play a role in peripheral nerve regeneration after injury. To this purpose, gold (Au), silver (Ag), and copper (Cu) can be used. Whether combined with hydrogels in scaffold materials, they are responsible for conduit topography modification, mechanical strength improvement, enhancement in neurotrophic factor secretion, and amelioration in the ion flow, as well as in the electrical signals’ regulation [86]. Among metal nanoparticles, Au nanoparticles are the most stable, also showing unique electrical, optical, and magnetic properties that make nerve conduits enriched with them a sort of therapeutic device [87]. In vitro studies on immature neuronal cell line proved their ability to stimulate cell adhesion, proliferation, and differentiation, also stimulating axonal elongation, and sprouting axons [88]. As reported by Lin et al. [89] they could ameliorate axonal growth and myelination, as well as prevent neuronal death.

## 5. Cells

Combining Schwann cells or immuneprivileged mesenchymal stem cells (bone marrow-derived and adipose-derived stem cells) with nerve conduits represents a promising therapeutic strategy in animal models of PNI [90,91], creating a favorable environment for nerve regeneration [92].

Schwann cells have been recognized as the major regenerative source of the peripheral nervous system due to their role in the normal repair response, where they adjust their physiology to support and control neuronal function through the release of neurotrophic factors (among all, nerve growth factor (NGF), brain-derived neurotrophic factor (BDNF), neurotrophin-3 (Nt3), ciliary neurotrophic factor (CNTF), and glial cell-derived neurotrophic factor (GCNF)) [93]; these molecules, together with adenosine triphosphate (ATP) and neuregulin released from the proximal stump, trigger the formation of new Schwann cells and, together with acetylcholine, stimulate their further proliferation [94]. In consideration of this, transplanted Schwann cells can boost nerve repair; preclinical studies highlighted that Schwann-cells filled conduits led to myelinated axons with larger diameters and thicker myelination than conduits without cells [95,96].

Bone marrow stromal cells are multipotent somatic stem cells, from a nonhematopoietic precursor, that can differentiate in mesodermal cell linages and neural phenotypes, including Schwann-like cells. Whether cultured in an adequate culture media, they transdifferentiate, then show a glial cell-like phenotype expressing the specific markers S100, Glial Fibrillary Acidic Protein (GFAP), and p75. In vitro differentiated cells lead to promising results when associated with artificial conduits and acellular grafts, promoting axonal and functional recovery [90]. Within the conduit they are recognized to favor cells’ homing ability and neurotrophic factor secretion, also differentiating into Schwann-like cells [97].

Adipose stem cells can be isolated in great numbers (one gram of adipose tissue can yield 3.5 × 10^5^ to 1 × 10^6^ stem cells; one gram of bone marrow can only yield 500 to 5 × 10^4^ stem cells [98]), and with minimally invasive methods (differently from bone marrow stem cells) from patient’s own adipose tissue. They secrete several neurotrophic factors, also upregulating this secretion by Schwann cells. In turn, an improved myelination and regeneration with a decreased nerve fibrosis can be observed, whether they are used to treat PNI, as shown through animal models of disease treated by direct repair, nerve grafting, and positioning of nerve conduits or nerve allografts [99]. Di Summa et al. [92] demonstrated that fibrin conduits enriched with adipose stem cells previously differentiated towards a Schwann cell-like phenotype allowed a greater axonal regeneration than empty conduits did. Unfortunately, the differentiation protocol can take more than 2.5 weeks, prolonging denervation and thus compromising functional recovery. To overcome this limitation, recurring to undifferentiated adipose stem cells could be appealing. The mechanisms through which they act are still not well established but, possibly, the secretion of neurotrophic, neuroprotective, and anti-inflammatory factors is involved. As broadly discussed by Zhang and Rosen [100], elucidating the mechanisms, side effects, and efficacy of undifferentiated adipose stem-cell-based nerve regeneration is required.

## 6. Conclusions

The current management of PNI is not fully satisfactory and, to date, there is a broad consensus that further progress in the field of peripheral nerve regeneration and success in clinical practice will no longer be dependent on vanguard and complex microsurgical tools and techniques or simple hollow nerve guide conduits. Instead, the scientific community agrees that the best way to move forward is through the development of next-generation devices based on merging the specific knowledge of different disciplines to guarantee a high innovation level [101]. Within this scenario, applying the principles of Tissue Engineering may have appealing results [102]; to guide the natural process of nerve regeneration, topographical/mechanical, biochemical (conductive guides, growth factors/peptides), and biological (support cells such as Schwann cells) cues have to act synergistically to provide a favorable environment for neural tissue regeneration [103]. To achieve that, different manufacturing methods, including dip coating, low-temperature deposition, lyophilization, solvent casting, electrospinning, solvent leaching, inject 3D printing, and extrusion-based printing have been adopted to fabricate nerve conduits according to the specific chemical/physical characteristics of the different materials used [104,105]. However, advances in material science itself is also fundamental, as proved by the many material options that are standing out (ranging from biopolymers and synthetic polymers to blends), able to exploit the different and specific material properties, including mechanical behavior, biocompatibility, degradation profile, and the ability to be differently combined with growth factors and proteins of interest [58].

Mechanical/physical properties (degradation profile matching the nerve regeneration rate, tensile modulus, conduit size, wall thickness, fit to the stump) and surface chemistry/morphology (bioactivation with adhesion molecules, conductive behavior, micro/nanopatterning with linear geometries), are the first goals to aim for to assure an efficient nerve conduit fabrication. Moreover, “cues” loading (neurotrophic factors, proteins, and anisotropy molecules) is advantageous [58]. Hence, ideally, the fabrication of a transplantable “living” platform is the ambitious goal to pursue. In this field, the new polymer, oxidized polyvinyl alcohol (OxPVA), seems to be extremely promising, responding to all the requirements above. This material represents a sort of evolution from the non-resorbable polyvinyl alcohol, of which the commercially available SaluBridge^TM^/SaluTunnel^TM^ (Salumedica LLC) guides are made. With respect to its native counterpart, OxPVA is distinguished by a tunable biodegradation rate, highly potential bioactivity, and adjustable physical characteristics, thus standing out as a customizable polymer, as proved by the ability to satisfy different prosthesis characteristics for different tissue targets [36,69,70,102,106,107,108,109,110,111]. Additionally, the possibility to be “impressed” or poured with/within specific molds and safely physically cross-linked (freezing-thawing method) without potentially toxic chemical agents is also fundamental from the perspective of creating “cell-instructive devices” (such as those encouraging an orderly regeneration of the axons) with no cytotoxic effect [69]. A successful functionalization of the polymer-derived supports can also be easily obtained by developing bilayer scaffolds (OxPVA + decellularized extracellular matrix/bioactive sheath) [70,108], incorporating cues (mechanical incorporation before cross-linking) [69], and adsorbing neurotrophic growth factors for in situ release, thus creating drug-delivery systems [102,107]. OxPVA hydrogel’s excellent mechanical properties are highlighted by their suture retention, ability to adapt the stump caliber, and lack of kinking in derived conduits [36]. Although there is the need for further studies, the premises are promising.

Together with intense research and development in material science as well as in new conduit fabrication, a critical reflection must be addressed to device effectiveness evaluation: beyond in vitro studies, the guides for PNI recovery need to be verified through preclinical animal models able to reflect the regeneration mechanisms that occur following human peripheral nerve injury. Currently, no animal species among those considered in published studies (from high to low frequency of use: rats, mice, rabbits, dogs, cats, sheep, monkeys, and pigs) respond to all the requirements for an ideal animal model, including the possibility to evaluate regenerative neurobiology aspects and pre-clinical efficacy, contextually. Moreover, according to the animal species and to the surgery site (sciatic, peroneal, tibial, facial, median, radial, ulnar, alveolar, cavernous, saphenous, and hypogastric), different lesion gap lengths (rat: up to 50 mm; mice: up to 13 mm; rabbit: up to 50 mm; dog: up to 90 mm; cat: up to 50 mm; monkey: up to 50 mm; pig: up to 8 mm) are reported, as well as different follow-up and endpoints that make comparison studies based on data from the Literature difficult [112,113]. The identification of appropriate animal models, and their limitations and benefits, is mandatory to achieve reliable pre-clinical data [114], thus guiding optimized conduit design before translating such technologies from bench to bedside [115].

## Figures and Tables

**Table 1:** US Food and Drug Administration (FDA) approved nerve conduits for clinical use.

Commercial Device	Manufacturer	Composition	Gap	Degradation Time	Maximum Length; Inner Diameter	Device ID, Year of FDA Approval
NeuroTube^TM^	Synovis Micro Companies Alliance, Inc., Birmingham, AL, USA	PGA	2.0–4.0 cm	3 months	30 mm; 2–8 mm	K983007, 1999
SaluBridge^TM^	Salumedica LLC, Atlanta, GA, USA	PVA	4.0–6.35 cm	Not resorbable	40 mm; --	K002098, 2000
NeuraGen^TM^	Integra LifeSciences, Princeton, NJ, USA	Collagen type I + glycosaminoglycan (chondroitin-6-sulfate)	0.5–1.7 cm	4 years	20–30 mm; 15–30 mm	K011168, 2001
NeuroMatrix^TM^	Collagen Matrix, Inc., Franklin Lakes, NJ, USA	Collagen type I	2.5–3.0 cm	4–8 months	25 mm; 2–6 mm	K012814, 2001
SaluTunnel^TM^	Salumedica LLC, Atlanta, GA, USA	PVA	4.0–6.35 cm	Not resorbable	40 mm; --	K100382, 2010
Neurolac^TM^	Polyganics Inc., Groningen, The Netherlands	D,L-PLCL	Up to 2.0 cm	16 months	25 mm; N/A	K050573, 2005 K112267, 2011
NeuroMend^TM^	Collagen Matrix, Inc., Franklin Lakes, NJ, USA	Collagen type I	0.9–2.5 cm	4–8 months	25–50 mm; 4–12 mm	K060952, 2006
Avance^®^ Nerve Graft	Axogen, Inc. Alachua, FL, USA	Decellularized cadaveric nerve	2.5–3.0 cm	--	15–70 mm; 3–5 mm	2007
NeuroFlex^TM^	Collagen Matrix, Inc., Franklin Lakes, NJ, USA	Collagen type I	2.5 cm	4–8 months	25 mm; 2–6 mm	K131541, 2014
NeuraGen^TM^ 3D Matrix	Integra LifeSciences, Princeton, NJ, USA	Collagen type I + glycosaminoglycan (chondroitin-6-sulfate)	2.5 cm	4–8 months	20–30 mm; 1.5–7 mm	K011168, 2014
Reaxon^TM^ Plus	Medovent GmbH, Mainz, Germany	Chitosan	Up to 1.0 cm	Significant macroscopic signs of degradation after 74/77 weeks	14 mm; 2.1–6.0 mm	K143711, 2015
AxoGuard^TM^ Nerve Connector	AxoGen, Inc., Alachua, FL, USA	Porcine SIS (mainly collagen types I, III, IV, and VI)	0.5–1.0 cm	3–4 months	10 mm; 1.5–7 mm	K162741, 2016

D,L-PLCL, Poly(DL-lactide-ε-caprolactone); PGA, Polyglycolic acid; SIS, small intestinal submucosa; PVA, Polyvinyl alcohol.

## Data Availability

Data sharing is not applicable to this article as no new data were created or analyzed in this study.
